# Clinical Value of Circulating microRNAs in Diagnosis and Prognosis of Pancreatic Cancer: A Systematic Review

**DOI:** 10.7759/cureus.43931

**Published:** 2023-08-22

**Authors:** Mithum Senaratne, Shivling S Swami, Soe Lwin Aye, Yash Trivedi, Zoryana Bolgarina, Heet N Desai, Lubna Mohammed

**Affiliations:** 1 Internal Medicine, California Institute of Behavioral Neurosciences & Psychology, Fairfield, USA; 2 Obstetrics & Gynecology, California Institute of Behavioral Neurosciences & Psychology, Fairfield, USA

**Keywords:** microrna, pancreatic cancer, molecular biomarker, pancreatic cancer diagnosis, pancreatic cancer prognosis, highly sensitive biomarker, highly specific biomarker, ductal pancreatic carcinoma, mir, circulating exosomes

## Abstract

Pancreatic cancer (PC) is one of the most common cancers and has a high mortality rate due to high invasiveness and rapid progression. Microribonucleic acid (microRNA) plays an essential role in diagnosing PC in the early stages, which improves the five-year survival rate. This systematic review aims to highlight the different subtypes of serum and plasma microRNAs and panel-based assays of microRNAs and how they play a crucial role in the diagnosis and prognosis of PC as a high-sensitive and specific novel biomarker. Following the Preferred Reporting Items for Systematic Review and Meta-Analysis (PRISMA) 2020 guidelines, an in-depth search was performed by using regular keywords and major Medical Subject Heading (MeSH) keywords in PubMed (MEDLINE), PubMed Central, Google Scholar, Science Direct, and Cochrane Library for articles related to this topic and published between 2013 and 2023, up to April 18, 2023. Further eligibility criteria and quality assessment tools were employed to assess the risk of bias, and 13 articles were finalized to be used in this review. The chosen articles included five cross-sectional studies, six systematic reviews and meta-analyses, and two literature reviews. This review provides strong evidence of the usage of microRNA for early diagnosis. It can also be used to exclude differential diagnoses of other diseases, and its prognostic value for determining metastasis and therapeutic efficacy in PC patients. Also, combining microRNA panels with carbohydrate antigen 19.9 (CA19-9) improves the sensitivity and specificity of microRNA as a biomarker.

## Introduction and background

Pancreatic cancer (PC) is the fourth most significant cause of cancer-related death in Western countries, and it is notorious for being aggressive, having a five-year survival rate of less than 10% [1]. According to the American Cancer Society (ACS) in 2017, there were 447,665 new cases of PC reported globally (58.6 per million), with a worldwide prevalence of 49.8 per million and 441,083 fatalities (57.7 per million) [2,3]. According to the National Cancer Institute (NCI), 64,050 new cases of PC were recorded, which is 3.3% of all new cancer cases; the estimated number of deaths in 2023 is 50,550 and this is 8.3% of all cancer death in the United States (US) in 2023 [4]. It’s estimated that, by 2025, death from PC may become the third leading cause of death, and in 2030, it will be the second leading cause of death, after lung cancer in the US [5,6].

These statistical data show two types of malignant tumors of the pancreas which can be divided into endocrine and exocrine malignancies: (i) Pancreatic adenocarcinoma, which is the most frequent histopathological exocrine malignancy and has morphological variants such as adenosquamous carcinomas, signet ring cell carcinomas, colloid/mucinous carcinomas, undifferentiated/anaplastic carcinomas, medullary carcinomas, and hepatoid carcinomas, and (ii) Non-ductal carcinomas, which are a type of endocrine malignancy and have various morphologies such as acinar cell carcinomas and neuroendocrine neoplasms [2,3]. The five-year survival rate of a PC largely depends on the diagnostic stage of the PC [2,3,7]. PC is very invasive, progresses rapidly, and remains asymptomatic for a long time due to its anatomical location until it reached advanced and infrequently incurable stages [2,3,7]. The five-year survival rates of those with localized (stage 0, I, and IIA) or regional (stage IIB and III) cancers are approximately 34% and 12%, respectively, and for PC patients with metastatic disease (stage IV) at the time of diagnosis, the five-year survival rate is 3.2% [4,8].

**Figure 1:**
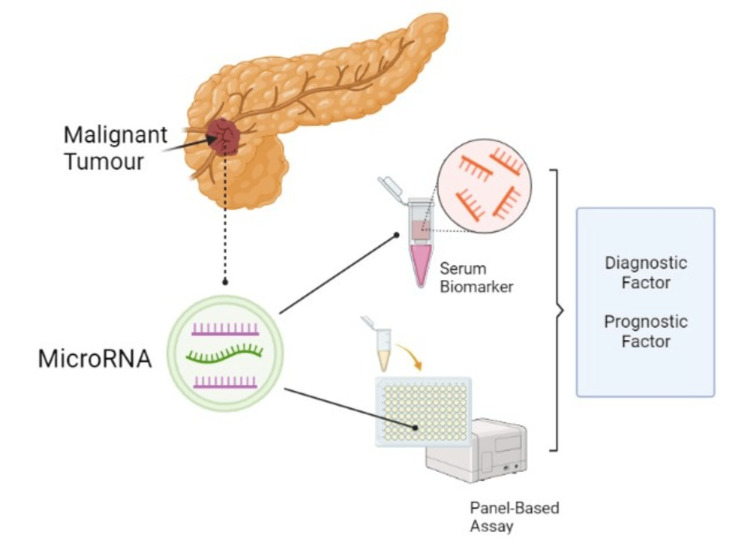
Presentation of microRNA as a diagnostic and prognostic factor in pancreatic cancer Image credit: Mithum Senaratne

Screening for PC in asymptomatic individuals at present is not recommended by the US Preventive Services Task Force (USPSTF) [9]. But the early diagnosis of PC is crucial for improving treatment outcomes. This can be done by techniques that are less accurate like magnetic resonance imaging (MRI), ultrasound, computed tomography (CT), and endoscopic ultrasonography (EUS) with EUS-guided fine-needle aspiration (EUS-FNA) [10]. Pancreaticoduodenectomy with additional chemotherapy is the only curative pathway; however, important complications make the therapeutic team’s effort difficult [11]. There has been attention on finding new diagnostic modalities or biomarkers that can help physicians discover PC early with high accuracy because of the predominant proportion of patients having unrespectable conditions at the time of diagnosis [12]. About 300 diagnostic biomarkers for PC are found in serum, biliary fluid, pancreatic juice, urine, feces, and pancreatic cystic fluid. Research into the effectiveness of microRNA as a diagnostic biomarker in PC has advanced significantly in the last decade due to the lack of a gold-standard diagnostic biomarker for PC [12]. microRNAs are a class of short non-coding endogenous RNAs found in cerebrospinal fluid, breast milk, saliva, and urine in addition to the serum that controls a number of physiological and developmental processes, including cell division, growth, death, and angiogenic differentiation [13-15]. Extensive research has recently demonstrated the pivotal function of microRNAs and panel-based assays of microRNAs, which is a very attractive methodology for microRNA detection in the onset, invasion, and metastasis of pancreatic cancer, has crucial implications for the diagnosis, prognosis, and treatment of cancer (Figure 1) [12-15]. microRNAs are now widely used in oncology for the diagnosis, prognosis, and monitoring of cancer treatment because of their promise as a diagnostic biomarker, having a wide number of prospective microRNA biomarkers in serum, and their capacity to endure adverse conditions and long-term storage [12,14,15]. There are two types of microRNA detection for the diagnosis and prognosis of pancreatic cancer: (i) single microRNA detection and (ii) microRNA assessing panels [12-15].

This formed the basis of our research question, “Do microRNA work as a potential biomarker in PC patients?''. Since 2010, many studies on the application of circulation microRNAs for the diagnosis of PC have been published. This systematic review aims to analyze and evaluate the available literature, especially from different subtypes of serum and plasma microRNAs and panel-based assays of microRNAs, and their importance as a diagnostic or prognostic tool that has high sensitivity and specificity for PC.

## Review

Method

The Preferred Reporting Items for Systematic Reviews and Meta-Analyses (PRISMA) 2020 guidelines [16] were followed for this systematic review. Removal of duplicate articles, identification, screening, and organization of full text according to inclusion and exclusion criteria were done manually and also with the help of Rayyan software (Rayyan Systems Inc., Cambridge, Massachusetts, US) [17].

Eligibility Criteria

Studies with the following criteria were selected as eligible for the review: studies written in the English language, studies conducted in any country, human studies (both male and female), adult age group (age>18 years), and free full-text articles published between 2013 and 2023. Further inclusion criteria encompass studies focused on using serum and plasma microRNA as a diagnostic or prognostic tool with high sensitivity and specificity biomarkers in individuals with PC. Animal studies, book chapters, conference papers, translated papers, editorials, protocols, only abstracts, and grey literature were excluded. Further exclusion criteria included paid articles, studies published before 2013, studies conducted in languages other than English, studies with patients aged <18 years, studies that focused on benign pancreatic tumors, pancreatitis, and other gastrointestinal cancers, and studies not included serum or plasma microRNAs.

Databases and Search Strategy

A search was conducted through PubMed (MEDLINE), PubMed Central, Google Scholar, Science Direct, and Cochrane Library. Keywords and Medical Subject Heading (MeSH) terms were used to identify all potentially relevant articles discussing the use of MicroRNA as a diagnostic or prognostic tool with high sensitivity and specificity biomarkers in individuals with PC. The search was performed using keywords such as microRNA, miRNA, miR, microRNA, Pancreatic Cancer, Pancreatic Carcinoma, Pancreatic ductal adenocarcinoma (PDAC), PC, Biomarker, Diagnosis, Prognosis, Sensitivity, and Specificity. The Boolean method was used to combine the keywords and MeSH terms to search through the various databases. Table 1 gives the details of the search strategy that was used in this systematic review.

**Table 1:** Details of the search strategy used in this systematic review.

Database	Search Strategy	Filters applied	Results
PubMed	(("microrna"[Title/Abstract] OR "miRNA"[Title/Abstract] OR "miR"[Title/Abstract] OR "microrna"[Title/Abstract] OR ("micrornas/analysis"[MeSH Major Topic] OR "micrornas/blood"[MeSH Major Topic] OR "micrornas/supply and distribution"[MeSH Major Topic])) AND ("Pancreatic Cancer"[Title/Abstract] OR "Pancreatic Carcinoma"[Title/Abstract] OR "Pancreatic ductal adenocarcinoma"[Title/Abstract] OR "PC"[Title/Abstract] OR ("pancreatic neoplasms/diagnosis"[MeSH Major Topic] OR "pancreatic neoplasms/diagnostic imaging"[MeSH Major Topic] OR "pancreatic neoplasms/prevention and control"[MeSH Major Topic] OR "pancreatic neoplasms/therapy"[MeSH Major Topic])) AND ("Biomarker"[Title/Abstract] OR "Diagnosis"[Title/Abstract] OR "Prognosis"[Title/Abstract] OR "Sensitivity"[Title/Abstract] OR "Specificity"[Title/Abstract] OR "biomarkers, tumor/analysis"[MeSH Major Topic] OR "biomarkers, tumor/blood"[MeSH Major Topic] OR "biomarkers, tumor/isolation and purification"[MeSH Major Topic])) AND ((y_10[Filter]) AND (ffrft[Filter]) AND (humans[Filter]) AND (english[Filter]))	Free full-text, Last 10 years, Humans, English, and Keywords only present in Title and Abstract	490
PubMed Central	((microRNA[Title] OR miRNA[Title] OR miR[Title] OR MicroRNA)[Title] AND (“Pancreatic Cancer”[Title] OR “Pancreatic Carcinoma”[Title] OR “Pancreatic ductal adenocarcinoma”[Title] OR PC)[Title] AND (Biomarker[Title] OR Diagnosis[Title] OR Prognosis[Title] OR Sensitivity[Title] OR Specificity)[Title]) Filters: published in the last 10 years	Last 10 years, Keywords are only present in the title.	667
Google Scholar	allintitle: (microRNA OR miR) AND (“Pancreatic Cancer” OR “Pancreatic Carcinoma”) AND (Biomarker OR Diagnosis OR Prognosis OR Sensitivity OR Specificity)	Last 10 years, Keywords only present in the title.	100
Science Direct	(microRNA OR miR) AND (“Pancreatic Cancer” OR “Pancreatic Carcinoma”) AND (Biomarker OR Diagnosis OR Prognosis OR Sensitivity OR Specificity)	Last 10 years, Keywords only present in Title and Abstract.	171
Cochrane Library	(microRNA OR miR) AND (“Pancreatic Cancer” OR “Pancreatic Carcinoma”) AND (Biomarker OR Diagnosis OR Prognosis OR Sensitivity OR Specificity)	Last 10 years, Keywords only present in the Title and Abstracts	07

Results

Study Selection and Quality Assessment

We identified a total of 1435 articles after applying our various search strategies across the five databases. We identified 490 articles from PubMed (MEDLINE), 667 articles from PubMed Central, 100 articles from Google Scholar, 171 articles from Science Direct, and seven articles from the Cochrane Library. A total of 82 duplicates were found and removed manually. The remaining 1353 articles were thoroughly searched and evaluated for relevance using the titles and abstracts. A total of 1310 articles were found to be irrelevant and were removed. Subsequently, 43 articles were sought for the retrieval of full texts. A further 28 articles were removed as they did not fulfill our inclusion/exclusion criteria.

The remaining 15 publications were individually evaluated for quality by SM and AS using study-specific techniques. Each assessment instrument has its scoring methodology and studies with a score of more than 70% were accepted for inclusion in this study. The quality evaluation of the studies, as well as the tools utilized, are summarized in Table 2. A further two articles were removed as they were deemed to be of lower quality (<70%) as per our criteria. A total of 13 articles were thus included in our study; of these, five were cross-sectional studies, six were systematic reviews and meta-analyses, and two were literature reviews. The PRISMA flow chart is provided in Figure ​2.

**Table 2:** Details of the quality assessment and the tools used for the final articles accepted for this review AXIS: Appraisal tool for cross-sectional studies; SANRA: Scale for the assessment of narrative review articles; AMSTAR: Assessment of multiple systematic reviews

Quality Assessment Tool	Type of Study	Total Score	Accepted Score (>70%)	Number of Accepted Studies
AXIS	Cross-Sectional study	20	>15	Guo et al., 2021 [18], Akamatsu et al., 2016 [19], Khan et al., 2021 [20], Vychytilova-Faltejskova et al., 2015 [21], Vila-Navarro et al., 2019 [22].
AMSTAR	Systematic Review, Meta-Analysis	16	>12	Wei et al., 2018 [23], Ye et al., 2019 [24], Hu et al., 2016 [25], Sun et al., 2018 [26], Peng et al., 2021 [27], Zhao et al., 2020 [28].
SANRA	Narrative Review	12	>9	Wnuk et al., 2023 [29], Tarasiuk et al., 2021 [30].

**Figure 2:**
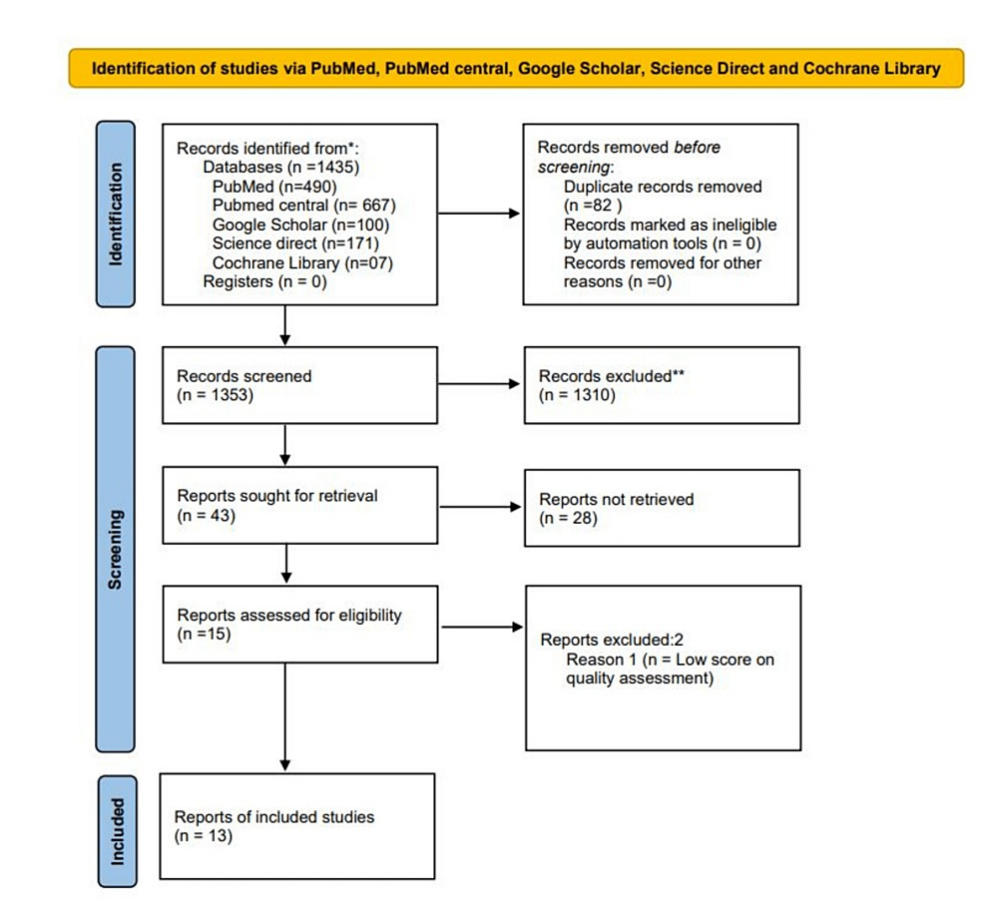
The PRISMA chart demonstrating the selection process of the included articles PRISMA: Preferred Reporting Items for Systematic Reviews and Meta-Analysis

Study Characteristics

The main study characteristics of each selected study are shown in Table 3.

**Table 3:** Main study characteristics of selected studies accepted in this review PC: pancreatic cancer; AUC: area under the curve; PDAC: pancreatic ductal adenocarcinoma; CP: chronic pancreatitis; AP: acute pancreatitis; EV: extracellular vesicles; EV: extracellular vesicles; AIP: auto-immune pancreatitis; MAPK: mitogen-activated protein kinase; HC: healthy control; CA 19-9: carbohydrate antigen 19.9; TP: true positive; FP: false positive; TN: true negative; FN: false negative; OS: overall survival

Author and year	Study Type	Inclusion and Exclusion criteria	Sample size/Number of studies	Outcomes and key points
Guo et al., 2021 [18]	Cross-Sectional Study	Inclusion Criteria: Consent for a blood specimen, diagnosed patients for PDAC & CP. Exclusion Criteria: Patients who underwent chemotherapy or radiotherapy before blood collection, patients with benign or borderline malignant tumors, patients with pancreatic metastases, pancreatic serous cystadenoma carcinoma, or mucinous cystadenocarcinoma, CP patients who had AP in the past three months, CP patients with malignant tendency follow-up period of six months, blood specimens with hemolysis levels greater than five.	90 Patients	Blood small EV microRNA can be used as a potential promising biomarker to aid in the differential diagnosis and prognosis of PDAC metastasis.
Akamatsu et al., 2016 [19]	Cross-Sectional Study	Inclusion Criteria: Diagnosed patients with PDAC, IPMN, AIP IPMN are confirmed adenomas, serum samples were obtained before the start of treatment. Exclusion Criteria: Patients who start treatments, diagnostically not confirmed patients.	69 patients	MAPK-associated microRNA in serum have the potential as a novel noninvasive biomarker for distinguishing PDAC from AIP.
Khan et al., 2021 [20]	Cross-Sectional Study	Inclusion Criteria: Diagnosed patients with PDAC and CP, the serum samples were processed and stored at -80 °C. Exclusion Criteria: Diagnostically not confirmed patients.	125 patients	Identified five serum microRNAs that could distinguish PDAC cases from CP and healthy controls with a high level of precision
Vychytilova-Faltejskova et al., 2015 [21]	Cross-Sectional Study	Inclusion Criteria: Specimens collected from patients who have undergone surgery. Exclusion Criteria: Patients are not diagnostically identified.	74 patients	Expression levels of microRNA-21, microRNA-34a, and microRNA-198 were characteristically higher; levels of microRNA-217 were considerably lower in PDAC compared to HC and CP. microRNA-21 and microRNA-198 were notably associated with shorter disease-free survival and overall survival.
Vila-Navarro et al., 2019 [22]	Cross-Sectional Study	Inclusion Criteria: Diagnosed patients with PDAC, IPMN, and CP, specimens were collected from peripheral venous blood only. Exclusion Criteria: Presence of hemolysis, having low microRNA extraction efficiency as detected by spiked-in cel-microRNA-39 levels.	190 patients	Identified altered circulating microRNAs that help to early detection of PDAC and found the highest diagnostic value tool as a combination of two microRNAs with CA 19-9
Wei et al., 2018 [23]	Systematic Review and Meta-Analysis	Inclusion Criteria: Related to the diagnostic value of miRNAs for PC, microRNA expression levels were discovered in serum or plasma, all patients were diagnosed as PC by using the gold standard test, and sufficient data were provided to calculate estimates of TP, FP, TN, and FN. Exclusion Criteria: Unrelated to the diagnostic value of microRNAs for PC, redundant publications or insufficient data letters, reviews, case reports, and editorials, studies not carried out on humans.	27 Studies	Serum- or plasma-based microRNAs are capable of distinguishing PC from non-PC with relatively high sensitivity and specificity.
Ye et al., 2019 [24]	Systematic Review and Meta-Analysis	Inclusion Criteria: The articles must be about pancreatic cancer, the microRNA must be present in the exosomes, the article must explore the relationship between exosomal microRNAs and pancreatic cancer, sensitivity, specificity, and AUC. Exclusion Criteria: Mechanistic studies, reviews studies without controls, the article without sensitivity, specificity, or AUC.	9 Studies	Multiple Exo-microRNAs were utilized concurrently, but increasing sensitivities at relatively low specificity levels can be obtained by particular microRNA combinations.
Hu et al. 2016 [25]	Systematic Review and Meta-Analysis	Inclusion Criteria: Studied PDAC based on histopathological verification, expression of microRNA-21 was calculated, and the association between the expression of microRNA-21 level and survival outcome was reported. Exclusion Criteria: Reviews, letters or laboratory studies, unpublished studies with only the abstracts, presented at national and international meetings, studies had overlapping or duplicate data, absence of adequate data to calculate the hazard ratio.	12 Studies	identified that increased microRNA-21 expression levels can predict poor prognosis in patients with PDAC
Sun et al., 2018 [26]	Systematic Review and Meta-Analysis	Inclusion Criteria: Studies highlighting the diagnostic value of microRNAs in PC, sufficient information is being reported to constructal a four-fold contingency table, published in the English language and Chinese language. Exclusion Criteria: Duplicate publications reviews, abstracts, letters, comments, and case reports Incomplete data to construct a four-fold contingency table, animal studies, and other basic research, number of samples in each group is less than 10.	40 Studies	microRNA had a substantial influence on PC diagnosis with high sensitivity and specificity, especially when several microRNAs are used
Peng et al., 2021 [27]	Systematic Review and Meta-Analysis	-	46 Studies	Circulating microRNAs demonstrated adequate diagnostic performance for PC, including early-stage PC, and the combination of circulating microRNAs with CA19-9 can increase diagnostic accuracy even further, giving a unique technique for PC diagnosis.
Zhao et al., 2020 [28]	Systematic Review and Meta-Analysis	Inclusion Criteria: Articles on the connection between microRNA expression level and survival time of PC patients, the inclusion of targeted OS outcomes, full text in English. Exclusion Criteria: Articles without original data (reviews, letters, or laboratory studies), no dichotomous microRNA level, the number of research examining the OS of microRNA expression levels equal to or less than two in tissue.	57 Studies	Identified blood microRNA-21, microRNA-451a, and microRNA-1290 have significant prognostic value.
Wnuk et al., 2023 [29]	Narrative Review	-	65 Studies	To assess the clinical use of circulating microRNA in PDAC therapeutic screening, diagnosis, prognosis, and monitoring.
Tarasiuk et al., 2021 [30]	Narrative Review	-	-	To assess current progress in the development and characterization of novel biomarkers, microRNAs, that may be beneficial in the early diagnosis of PDAC.

Discussion

Conventional Methods Used in PC Diagnosis

PC is still one of the most difficult malignancies to treat, with a five-year survival rate of less than 5% [23,28]. It is highly aggressive; distant metastasis and tissue invasion may occur at initial stages [23,24,28]. Because of its strong resistance to chemotherapy and radiation, surgical excision is the most successful treatment. Early detection of PC is critical for enhancing treatment results, as early-stage resection can result in an additional two years of survival [23,24,28,29]. Radiological imaging techniques like MRI, CT, positron emission tomography (PET), and invasive techniques like endoscopic retrograde cholangiopancreatography (ERCP), EUS, and EUS-FNA are conventional methods used in PC diagnosis [23,24,26-29]. Because of its increased sensitivity and specificity, EUS must be regarded as the most accurate diagnostic method among these, and it simplifies the retrieval of specimens of suspicious tissue by EUS-FNA for pathological confirmation [10].

Invasive procedures are utilized after local and systemic symptoms manifest, leaving most PC patients at an advanced stage at the time of diagnosis [23,24,26,28,29]. In liquid biopsy, various substances released by the pancreatic tumor are detected [27]. Because of its noninvasiveness, better sensitivity, and cost-efficiency, liquid biopsy is of tremendous interest from both scientific and therapeutic perspectives. [27,29]. Circulating tumor cells (CTCs), circulating tumor extracellular vesicles (exosomes), cell-free circulating tumor DNAs (cDNAs), circulating microRNAs, long noncoding RNAs (lncRNAs), proteins and metabolites, tumor-educated platelets (TEPs), CA 19-9, CA-50, CA-125r, matrix metallopeptidase-9 (MMP-9), Kirsten rat sarcoma viral oncogene homolog (K-ras), tumor protein 53 (p53), and carcinoembryonic antigen (CEA) can be detected by liquid biopsies [27]. Thus, because of the various drawbacks like inadequate specificity and unreliable sensitivity in the other biomarkers, circulating microRNAs are more suitable liquid biopsy markers showing high sensitivity and specificity with minimally invasive procedures [23,24,26].

microRNAs

microRNAs are a group of small, non-coding, endogenous RNAs with 19-25 nucleotides that determine the expression of genes at the post-transcriptional level [20,22,23]. They alter the stability and translation of nuclear messenger RNA (mRNA) transcripts by preventing mRNA translation or identifying mRNAs for degradation by binding to their three-end (3') or five-end (5') untranslated region (UTR) or opening reading frames of the targeted mRNA [20,22,23]. They can function as oncogenes (oncomiRs) and tumor suppressors by controlling the translation of more than 60% of protein-coding genes [22,23,28]. They also significantly regulate a wide range of biological processes, including development, cellular proliferation, differentiation like epithelial-mesenchymal transition (EMT), and apoptosis [22,23,28]. The differential expression of microRNAs in normal and cancerous tissues can be caused by chromosomal changes due to DNA point mutations, epigenetic mechanisms, or changes to the microRNA processing machinery of the microRNA genes, which are frequently found in genomic regions linked to cancer [20-22].

These dysregulated microRNAs target one or even hundreds of mRNAs that can be implicated in regulating important pathological processes in tumor formation, advancement, and metastasis [21,22]. These highly produced microRNAs may enter the circulation from disease tissues such as PC and its precursor lesions as part of the extracellular interaction between cells that functions as a hormone-like signal [20,22,23]. Different microRNA categories can be used as biomarkers like single microRNAs, two microRNA panels, panels with ≥ three microRNAs, microRNA panels combined with CA 19-9, and microRNAs derived from serum exosomes [23,24,26].

microRNAs derived from serum exosomes can be divided into single patterns and exosomal microRNA (exo-miR) panels [23,24,26]. These different types of microRNAs and their regulation are shown in Table 4. Exosomes are double-layer phospholipid membrane vesicles secreted by most cell types (tumor cells can secrete 10 times as many exosomes as normal cells) with small 30-100nm diameters containing various proteins and nucleic acids remain the major source of circulating microRNA [23,24,30]. They activate receptors to mediate the neighboring or long-distance cell-cell communications as specific signaling molecules before they are released into a recipient cell, which can be detectable in serum [23,24,30]. Exo-miR may be novel biomarkers for PC screening and diagnosis because their concentration differs from tumor stages and stable molecules, which are protected from plasma and cellular RNAs [23,24].

**Table 4:** Different types of microRNA and their expression CA 19-9: carbohydrate antigen 19.9

Diagnostic Role		
microRNA category	Types of microRNA	Expression
Single microRNA	miR-155-5p, miR-16-5p, miR-181a-5p, miR-181b, miR-192-5p, miR-21-5p, miR-221-3p, let-7e-5p, let-7f-5p, miR-103a-3p, miR-151a-5p, miR-151b, miR-23-3p, miR-320a, miR-33a-3p, miR-548d-3p, miR-93 [21] miR-10b, miR-21, miR-30c, miR-106b, miR-20a, miR-181a, miR-483, miR-25, miR-34a, miR-22, miR-642, miR-885, miR-7, miR-181d, miR-193b, miR-223, miR-744, miR-155, miR-212, miR-192, miR-194, miR-196a, miR-196b, miR-182, miR-16, miR-17, miR-1290, miR-628, miR-550, miR-1825, miR-221, miR-210 [23].	Upregulated
	miR-let7a, miR-122,miR-373,miR-492,miR-663a [23].	Downregulated
Two microRNA panels	(miR-10b, miR-106b), (miR-192,miR-194) , (miR-16,miR-196a) [23] (miR-16, miR-196a) [26].	Upregulated
	(miR-492, miR-663a) [23].	Downregulated
Panels with ≥ 3 microRNAs	(miR-1246, miR-4644, miR-3976, miR-4306), (miR-885, miR-22, miR-642), (miR-20a, miR-21, miR-24, miR-25, miR-99a, miR-185, miR-191), (miR-21,miR-210,miR-155,miR-196a) [23]. (miR-21, miR-155, miR-216), (miR-22-3p, miR-642b-3p, miR-885-5p) [26].	Upregulated
	(miR-486, miR-126, miR-106b), (miR-486, miR-126, miR-106b, miR- 938, miR-26b, miR-1285) [23].	Downregulated
	(miR-16, miR-27a, miR-30a, miR-323, miR-20a, miR-29c, miR-483), (miR-16, miR-24, miR-27a, miR- 30a, miR-323, miR-20a, miR-25, miR-29c, miR-483), (miR-16, miR-27a, miR-25, miR-29c, miR-483),(miR-16, miR-18a, miR-24, miR-27a, miR-30a, miR-323, miR-20a,miR-25, miR-29c, miR-191, miR-345, miR-483) [23].	Not Identified
miRNA panels combined with CA 19-9	(miR-196, miR-200, CA 19–9), ( miR-182, CA 19–9), ( miR-16, CA 19–9), (miR-16, miR-197a, CA19–9) [23].	Upregulated
	(miR-16, miR-27a, miR-30a, miR-323, miR-20a, miR-29c, miR-483, CA 19–9), (miR-16, miR-24, miR-27a, miR-30a, miR-323, miR-20a, miR-25,miR-29c, miR-483, CA 19–9), (miR-16, miR-27a, miR-25, miR-29c, miR-483, CA 19–9), (miR-16, miR-18a, miR-24, miR-27a, miR-30a, miR-323, miR-20a,miR-25, miR-29c, miR-191, miR-345, miR-483, CA 19–9) [23].	Not Identified
microRNAs derived from serum exosomes	(miR-1246, miR-4644, miR-3976, miR-4306), (miR-17) [23], (miR-17-5p), (miR-21), (miR-196a, miR-1246), (miR-191), (miR-21), (miR-451a), (miR-122-5p, miR-193b-3p) [24].	Upregulated
		Downregulated
Exosomal microRNA panels	(miR-3976, miR-4306, miR-1246, miR-4644, miR-3976, miR-4306) [24].	Upregulated

The combination of biomarkers has been explored and may overcome the current limitations like lack of specificity, sensitivity, and suboptimal diagnostic performance of single exo-miR biomarkers [24]. A suitable combination of biomarkers should be able to distinguish between healthy participants and patients, which also can be expressed in the disease progression [30]. For example, exo-miR-122-5p or exo-miR-193b-3p alone has a low sensitivity and specificity for PC detection, but the sensitivity of the combined panel is significantly improved, and the specificity is at a reasonable level [24]. 

The clinical importance of microRNAs is connected to the diagnosis, staging, progression, prognosis, and therapeutic response of PC because they are very stable molecules that provide a serial analysis according to the changes in the disease state, are sensitive to degradation, have more reliable expression levels, and enable rapid collection through less invasive techniques [22,23,26].

Early Diagnosis of PC Using microRNA

The detection of high-grade dysplasia before its malignant transformation, the time and frequency of malignant development, and the detection of early invasive carcinoma are key challenges in the therapy of PC patients and crucial for patients' survival [22]. Intraductal papillary mucinous neoplasms (IPMNs), which are benign pancreatic cysts in the ducts of the pancreas, have a tendency to develop into malignant tumors, which are only detectable radiographically as PC precursor lesions [22]. Circulating microRNAs in plasma show a significantly different expression profile from non-neoplastic pancreatic lesions and stages of neoplastic lesions (IPMN or PDAC) suggesting that they may be used as noninvasive biomarkers for PC [22].

microRNAs have exhibited diagnostic efficacy in early-stage PC patients (stage 0-IIa) with high sensitivity and specificity [27]. Based on a prospective cross-sectional study by Vila-Navarro et al., two microRNAs (miR-181b-5p and miR-548d-3p) have been identified that are markedly elevated in PDAC plasmas but not in pre-malignant IPMNs, indicating that their late rise in levels may be connected to the malignant progression of IPMNs and may be specific to invasive or high-risk IPMNs, facilitating the differentiation between low- and high-risk IPMNs [22]. In their respective studies, Vila-Navarro et al. found that 17 circulating microRNAs were upregulated, Wei et al. found that 32 oncogenic miRNAs were upregulated, five anti-oncogenic microRNAs were downregulated, and Ye et al. found that nine serum single exo-miR upregulated [22-24]. miR-21 is the most common early diagnostic single microRNA is in PC and is more valuable than other microRNAs [21,23,24,26,29]; miR-155, miR-196, and miR-210 are also commonly used single microRNAs for early detection of pancreatic cancer [29]. A combination of miR-196a and 196b exhibited high diagnostic accuracy, with sensitivity and specificity values greater than 90% [27]. Serum exo-miR-1226-3p is regarded as a potential biomarker in early diagnosis and predicting the tumor invasion or metastases of PDAC [30]. Table 4 shows the expression of different microRNAs that facilitate the diagnosis of PC [22-24,26].

microRNA as a Differential Diagnostic Biomarker

Low accuracy of early diagnosis and misinterpretations of PC becomes a clinical challenge due to the relative absence of early symptoms and the difficulty to distinguish PC and other non-cancerous pancreatic diseases such as chronic pancreatitis, pancreatic cyst, autoimmune pancreatitis (AIP), and IPMN by using imaging techniques [18,25]. For instance, it is frequently difficult to distinguish between PDAC and chronic pancreatitis in the early stage of the disease using imaging techniques like high-resolution CT, MRI, and PET [18,20]. This leads to incorrect diagnoses, inappropriate treatments, and a critical need to improve patient outcomes because recurrent chronic pancreatitis may be a risk factor for PDAC [18,20].

Novel diagnostic biomarkers are therefore urgently needed to enable accurate differential diagnosis of pancreatic lesions [21]. The commonest histopathological subtype is pancreatic cancer is PDAC [29]. Guo et al. reported on using blood-small extracellular vesicle (EV) derived microRNAs to differentiate PDAC from chronic pancreatitis in clinical patients [18]. Small RNA sequencing was performed in this investigation to discover small blood EV miR-95-3p/miR-26b-5p as a potential diagnostic biomarker to distinguish PDAC from chronic pancreatitis patients using clinical blood samples from pathologically diagnosed patients [18]. Some small EV microRNAs, such as miR-10b, miR-21, miR-30c, miR-181a, and miR-let7a, showed differences in expression among PDAC patients, healthy controls, and chronic pancreatitis patients [18]. Khan et al. discovered two down-regulated microRNAs (miR30b5p and miR320b) and three up-regulated microRNAs (miR2155p, miR1225p, and miR1925p) in PDAC compared to chronic pancreatitis. The expression patterns of miR-215-5p, mir-122-5p, and miR-192-5p are gradually rising in PDAC compared to chronic pancreatitis. At the same time, the levels of miR-30b-5p in the serum steadily reduced from healthy controls to chronic pancreatitis to PDAC. As a result, tracking their serum levels during the chronic pancreatitis stage may be a valuable marker for the early identification of PDAC [20].

Vila-Navarro et al. expressed that six microRNAs (let-7e-5p, let-7f-5p, miR-103a-3p, miR-151a-5p, miR-221-3p, and miR-23a-3p) significantly altered in IPMN and PDAC but chronic pancreatitis had normal levels, suggesting that these six microRNAs could be more specific of pancreatic neoplasia [22]. The expression of serum exo-miR-1226-3p was deregulated in PDACs compared to pancreatic lesions, which are accepted to predict the tumor invasion or metastasis of PDAC [30]. AIP is a rare form of chronic pancreatitis that is a benign inflammatory disease that mimics PDAC both clinically and radiologically; thus, simple and minimally invasive alternative markers for differential diagnosis would be useful because prognosis and treatment differ significantly from those for PDAC [19]. 

Mitogen-activated protein kinase (MAPK) and associated microRNAs have been identified to have high specificity and sensitivity for differentiating PDAC from AIP by Akamatsu et al. [19]. Patients with PDAC or IPMN had significantly higher serum levels of microRNAs associated with MAPK than patients with AIP (p = 0.009 for miR-7, p = 0.002 for miR-34a, p = 0.001 for miR-181d, p = 0.002 for miR-193b), confirming the distinct microRNA expression between AIP, PDAC, and IPMN, and indicating that they might help with a more accurate diagnosis of PDAC [19]. Significantly increased levels of plasma small EV-miR-196a, miR-1246, miR-21, miR-34a, and miR-198, and decreased levels of miR-217 have been observed in PDAC samples compared to healthy individuals [18,21].

Also, exo-miR-17-5p and exo-miR-21 can identify PC from non-PC participants (including ampullary carcinoma, benign pancreatic tumor, chronic pancreatitis, and healthy participants) [24]. There are relevant microRNA diagnostic panels that can use to distinguish between patients with PC, patients with chronic pancreatitis, and healthy individuals. Two diagnostic microRNA panels to distinguish patients with PDAC from healthy individuals include: (miR-145, miR-150, miR-223, miR-636) and (miR-26b, miR-34a, miR-122, miR-126, miR-145, miR-150, miR-223, miR-505, miR-636, miR-885.5p) [30].

microRNA as a Prognostic Biomarker

Factors including late clinical presentation, aggressive local invasion, high metastatic potential, and resistance to chemotherapy or radiation are the main causes of PC's poor prognosis [25]. These difficulties encourage scientists to look for more effective treatments for PC, such as novel biomarkers that may distinguish between phenotypes with varied clinical characteristics and prognoses [25].

Metastasis prognostic biomarker: The blood small EV-miR-335-5p and miR-340-5p were shown to be able to distinguish between metastasizing and non-metastasizing PDACs, which is crucial for determining PC survival [18]. The expression of miR-181d is significantly downregulated between patients with metastatic PC (stages III and IVb), supporting the association between serum miR-181d and metastasis in PDA patients (p = 0.014) [24]. Exo-miR-21, exo-miR-451a (the best prognosis biomarker), exo-miR-4525, and exo-miR-17-5p have been reported to vary from early-stage PC patients to advanced stages significantly; however, there are no associations for exo-miR-21 across tumor stages [24]. Clinical management of PDAC is favored by increased expression of miR-21 in the serum sample but it also emerged as an important predictor of poor prognosis in PDAC, indicating a stable and useful prognostic biomarker in PC [25,26,28,29]. The same effect was reported in miR-196a [29]. Male gender, positive vascular infiltration, and positive neural infiltration of the patient are not related with high miR-21 expression, while positive lymph node metastases demonstrate an important association, and when lymph node metastasis occurs, PC cells increase their ability to invade the body [25]. Zhao et al. reported that miR-21 and miR-451 are strong blood biomarkers for PC [28]. A panel consisting of downregulated miR-99a-5p, miR-365a-3p, and up-regulated miR-200c-3p has been proven to identify patients with poor prognosis after surgical resection, and two up-regulated microRNAs (miR-99a-5p and miR-125b-5p) are significantly associated with a shorter progression-free survival (PFS) [29]. Table 5 shows the expression of different microRNAs that have a prognostic role in PC [24-26,28].

**Table 5:** Different microRNAs and their prognostic ability miR: microRNA

Prognostic Role		
microRNA category	Types of microRNA	Expression
Single microRNA	miR-21, miR-451a ,and miR-1290 [24,25,27]	Upregulated
microRNA derives from serum exosomes	(miR-21), (miR-451a), (miR-4525) and (miR-451a) [23]	Upregulated

Prognostic biomarker for monitoring therapies: miR-21 expression is likely to be related to chemoresistance, and data shows that abnormal miR-21 expression severely impairs chemotherapy responsiveness, and increased miR-21 expression is also related to lower overall survival in chemotherapy patients [25]. The systematic review by Hu et al. showed that miR-21 expression is greater in PDAC cells with higher 50% inhibitory concentrations of gemcitabine or five-fluorouracil [25]. An increased plasma miR-21 and miR-744 expression indicates the tendency for worse PFS during gemcitabine therapy [29]. Furthermore, plasma miR-20a-5p expression has been seen to be considerably lower in gemcitabine-resistant PDAC patients compared to non-resistant patients. A high level of serum miR-373-3p prior to treatment and a low level of serum miR-194-5p after one cycle of FOLFIRINOX (folinic acid, fluorouracil, irinotecan, oxaliplatin) therapy were related to early tumor development. Plasma levels of miR-181a-5p are downregulated after treatment with FOLFIRINOX in non-progressive patients compared with progressive patients. Serum levels of miR-196a might be a useful marker to distinguish resectable and unresectable PDAC. miR-18a, miR-196a, miR-196b, miR-221, miR-223, miR-483-3p and miR-744 are downregulated after surgery for PC but miR-107 is upregulated after surgery [29].

microRNA as a High Sensitivity and Specificity Biomarker

CA19-9 is a tumor antigen identified in 1979 that has been used as a PC biomarker for decades [27]. The standard serum marker for monitoring response to therapy and predicting postoperative recurrence of PC is sialylated Lewis blood group antigen CA19-9, which has limited sensitivity and specificity for PDAC diagnosis [20,22,23,27]. It has few contraindications like being elevated in other malignancies like hepatocellular carcinoma, cholangiocarcinoma, and patients with non-malignant obstructive jaundice resulting in impaired specificity, often normal in precancerous masses patients and unsuitable for 10% of the White population who are unable to express CA19.9 because it is Lewis genotype negative [20,22,23,27].

**Figure 3:**
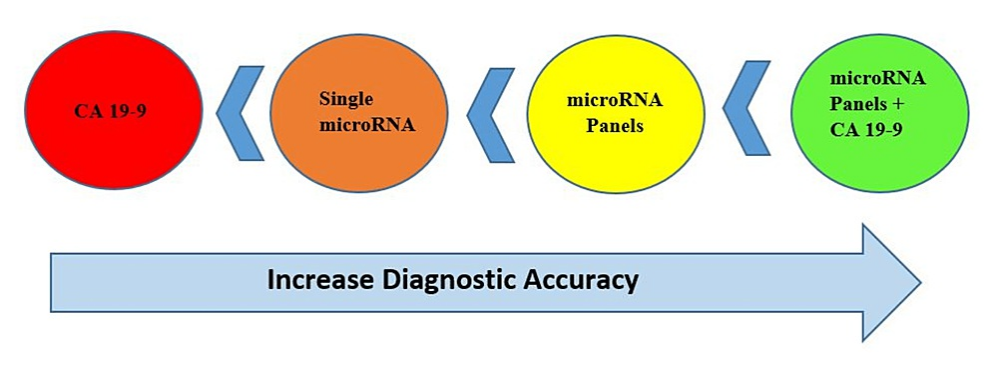
Presentation of the increasing diagnostic value of microRNA CA 19-9: carbohydrate antigen 19-9 Image credit: Mithum Senaratne

microRNA in PC diagnosis is still disputable and unsatisfactory because of the studies' extensive sensitivity and specificity values, which may cause different results depending on the subjects’ race, controls’ source, miRNAs’ types, and sample tested [26]. Single-circulating microRNAs are not specific to subgroups of gastrointestinal cancers because they are involved in various oncogenic pathways of different types of neoplasms [29]. To overcome this limitation of the diagnostic performance of single circulating microRNA, they were combined with panels and then combined with CA 19-9 [29]. These diagnostic panels reached higher diagnostic values than single circulating microRNAs shown in Figure 3 [18,22,27,29].

Based on the meta-analysis done by Sun et al., microRNAs have relatively high sensitivity and specificity in distinguishing patients from healthy controls, especially using multiple microRNA profiling (Table 4) with PC, which has a higher diagnostic value than traditional markers [26]. Peng et al. have discovered that the combination of circulating microRNAs and CA19-9 may be a novel and better strategy for diagnosing PC with more accuracy than circulating microRNA (single microRNA and microRNA panels) [27]. Guo et al. identified that the sensitivity was further enhanced when blood small EV-miR-95-3p/miR-26b-5p combined with serum levels of CA19-9 but the specificity was maintained at similar levels as the specificity of miR-95-3p/miR-26b-5p alone [18]. Vila-Navarro et al. described that the signature “miR-33a-3p1miR-320a” can differentiate between patients harboring malignant PC or premalignant IPMNs and healthy individuals with high accuracy, and the addition of CA19.9 (two miRNAs combined with CA19.9) increases this performance, suggesting that this combination could be a signific biomarker signature for non-invasive early detection of pancreatic neoplasia [22].

Limitation

This review has certain limitations, which should be worth mentioning to avoid bias. This study was limited to free full-text articles published in the last 10 years in English. We focused on using circulating microRNAs as a diagnostic or prognostic tool with high sensitivity and specificity in adult individuals with PC when selecting articles. We excluded research findings that discussed microRNAs that are present in our body other than in serum or plasma, such as cerebrospinal fluid, breast milk, saliva, biliary fluid, pancreatic fluid, urine, etc. Most of the findings of clinical trials were compared with pancreatic adenocarcinoma rather than other PC types. This review does not mention every function of the microRNA type in oncogenesis and tumor suppression.

## Conclusions

This review provides an idea of how microRNA is important clinically when it comes to the diagnosis and prognosis of PC. It has been proven that microRNA is a suitable biomarker with high sensitivity and specificity for the early detection of PC and excludes other differential diagnoses which can’t be excluded by using imaging techniques and other conventional methods of a PC diagnosis. And also how it’s important in the metastasis prognosis of PC and monitoring the therapeutic efficacy of individuals with PC. It has been seen that sensitivity and specificity of microRNA have been improved by the combination of CA19-9 tumor marker with microRNA panels.

Therefore, the main aim of this review was to elaborate on the clinical value of microRNA, which is a minimally invasive cancer marker, as a novel biomarker that can be used for the diagnosis and prognosis of PC effectively as an alternative and additional strategy to more invasive diagnostic methods currently available. Suggestions for future research include conducting more clinical trials on circulating microRNA profiles of different types of PC, using microRNA for the prognosis of metastasis of PC, and identifying functions of different microRNA more precisely in oncogenic, tumor formation, and tumor suppression in PC patients.
